# The War Cure

**Published:** 1881-07

**Authors:** J. B. Chandler


### THE WAR CURE.

BY J. B. CHANDLER.

In the earliest traces of human beings we find rude implements of war. Or rather implements which might be used for offense or defense as well as for the procuring of fuel or food; such as stone arrowheads and axes.

In another less remote period, the use of metals, copper and iron, for similar purposes, has been proven, and the authorities note continued improvement in these rude weapons as men advanced toward civilization.

The stone taken from the brook by David in its efficiency seemed to have worked a miracle, so far advanced had the art of war and the weapons in use become in the time of the Phillestines. Yet how little had they progressed towards the arms of the nineteenth century.

We could, had we space, trace with interest the gradual development of the peoples and their advance in the science and appurtenances of war (for war went hand in hand with that development), noting the inventions which seemed to emanate from the peculiar manner of living in the early ages, in walled cities, castles, etc.—rude implements depending largely upon the physical strength of the operators, the battering ram, the movable tower for overlooking the walls, engines for hurling huge stones, enormous mechanical bows, and other rude contrivances which, in their time, brought victory to their possessors.

Even after the discovery of gunpowder, it seems to have been reserved for our generation to develop its full capability. Compare the efficiency of a good marksman, armed with a "matchlock," loaded with the ammunition of its time, with that of the same man trained to the use of a Winchester repeating rifle of the present day. The first weapon capable of killing at thirty yards, and of being loaded and discharged say ten times in an hour, the other shooting, with accuracy, 300 yards and repeating ten times a minute!

Shakespeare, when he wrote of

> ——"Mortal engines, whose rude throats,
> The immortal Jove's dread clamors counterfeit,"

little dreamed of the huge "Columbiads" or the death dealing beauties of the nineteenth century, discharging continually, upon the turning of a crank, mowing down whole regiments as they come to the front.

Compare the rude "battering ram," swinging to a pole, vibrating under the power of running men, with the effect upon the same walls of a single ball from a rifled siege gun at three miles distance, handled by scientific gunners.

Think of the power held in control by the little glass ball with which the Czar of Russia was murdered. And yet, thousands of scientific minds are to-day bent upon the discovery of more advanced implements of war, and rich rewards await the successful one.

At the first glance this seems terrible; that in the advanced state of civilization of this century men should be seeking amidst the hidden secrets of science for the means of slaughtering their fellowmen in tens of thousands, instead of thousands, the point they have now reached. Nevertheless, no matter if his aim be only selfish, the man who shall invent the means for the total annihilation of an army at one blow will be a great public benefactor, for with the advent of his machine the enlistment of armies will cease. War will be a thing of the past. Arbitration will become a power to settle all disputes. Great generals will have to return to their ploughs or their tanneries, and statesmen will be made of material other than pothouse politicians; for with continued peace must come the development of the higher faculties of the people, and mind will rule without force. Then we shall have men entirely great, and, "Beneath the rule of men entirely great, the pen is mightier than the sword."

